# Reassessment of the genetic basis of natural rifampin resistance in the genus *Rickettsia*

**DOI:** 10.1002/mbo3.1431

**Published:** 2024-07-31

**Authors:** Julien Amoros, Noor Fattar, Marie Buysse, Meriem Louni, Joanne Bertaux, Didier Bouchon, Olivier Duron

**Affiliations:** ^1^ MIVEGEC, CNRS, IRD University of Montpellier Montpellier France; ^2^ EBI, CNRS University of Poitiers Poitiers France

**Keywords:** natural antibiotic resistance, *Rickettsia*, *rifampin*, RNA polymerase β subunit, *rpoB*

## Abstract

*Rickettsia*, a genus of obligate intracellular bacteria, includes species that cause significant human diseases. This study challenges previous claims that the Leucine‐973 residue in the RNA polymerase beta subunit is the primary determinant of rifampin resistance in *Rickettsia*. We investigated a previously untested *Rickettsia* species, *R. lusitaniae*, from the Transitional group and found it susceptible to rifampin, despite possessing the Leu‐973 residue. Interestingly, we observed the conservation of this residue in several rifampin‐susceptible species across most *Rickettsia* phylogenetic groups. Comparative genomics revealed potential alternative resistance mechanisms, including additional amino acid variants that could hinder rifampin binding and genes that could facilitate rifampin detoxification through efflux pumps. Importantly, the evolutionary history of *Rickettsia* genomes indicates that the emergence of natural rifampin resistance is phylogenetically constrained within the genus, originating from ancient genetic features shared among a unique set of closely related *Rickettsia* species. Phylogenetic patterns appear to be the most reliable predictors of natural rifampin resistance, which is confined to a distinct monophyletic subclade known as Massiliae. The distinctive features of the RNA polymerase beta subunit in certain untested *Rickettsia* species suggest that *R. raoultii*, *R. amblyommatis*, *R. gravesii*, and *R. kotlanii* may also be naturally rifampin‐resistant species.

## Graphical abstract

Contrary to previous studies, the natural resistance of *Rickettsia*, a genus of intracellular bacteria, to the antibiotic rifampin is not solely due to the Leu‐973 residue in the RNA polymerase β subunit.

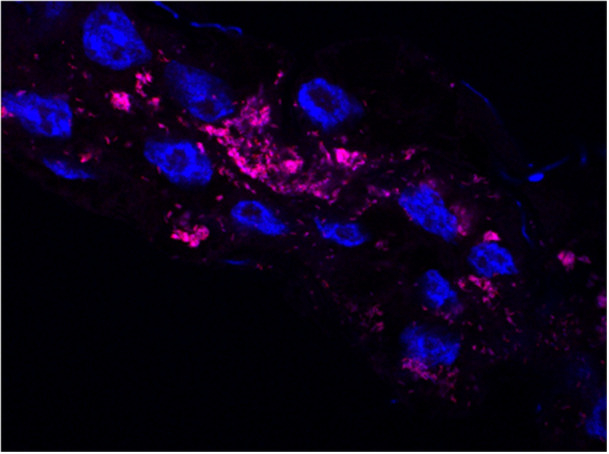

## 1 INTRODUCTION

Members of the *Rickettsia* genus are obligate intracellular bacteria that infect eukaryotes including humans and other vertebrates, arthropods but also protozoa, algae, and plants (Gillespie & Salje, 2023; Perlman et al., 2006; Weinert et al., 2009; Weinert, 2015). There are over 30 currently recognized *Rickettsia* species, classified into at least 10 distinct phylogenetic groups (Binetruy et al., 2020; Davison et al., 2022; Gillespie & Salje, 2023; Perlman et al., 2006; Weinert et al., 2009, 2015; Weinert, 2015). The most extensively studied species within this genus are those pathogenic causing human diseases, such as *R. prowazekii*, the causative agent of epidemic typhus, and *R. rickettsii*, responsible for Rocky Mountain spotted fever (Gillespie & Salje, 2023; Perlman et al., 2006; Weinert, 2015).

Rifampin (also termed rifampicin) is one commonly prescribed antibiotic for treating bacterial infections (Goldstein, 2014; Tupin et al., 2010), although it is not considered as a first‐line treatment for rickettsial infections in humans (Blanton, 2019). Rifampin is a broad‐spectrum antibiotic inhibiting bacterial RNA polymerase, thereby disrupting RNA synthesis and impeding bacterial protein production (Goldstein, 2014; Koch et al., 2014; Tupin et al., 2010). Rifampin is effective against certain *Rickettsia* species from diverse groups (Table 1). While limited data are available for the Transitional, Helvetica, Canadensis, and Bellii groups, rifampin resistance was more extensively investigated for species of the Spotted Fever and Typhus groups (Drancourt & Raoult, 1999; Rolain & Raoult, 2005; Rolain et al., 1998). Indeed, members of the Typhus group, such as *R. prowazekii* and *R. typhi*, are naturally sensitive to rifampin, as well as most species of the Spotted Fever group (Table 1). However, rifampin susceptibility is not a universal feature within the Spotted Fever group, as experimental studies have identified at least four naturally rifampin‐resistant species in this group: *R. massiliae*, *R. rhipicephali*, *R. montanensis*, and *R. aeschlimanii* (Eremeeva et al., 2006; Rolain et al., 1998, 2002).

**Table 1:** List of rifampin‐resistant (R) and susceptible (S) *Rickettsia* species in the literature

*Rickettsia* group	Species	Strain	GenBank accession number	Susceptibility to rifampin	Reference
Spotted Fever Group	*Rickettsia aeschlimannii*	MC16	GCA_001051325^a^	R	Rolain et al. (1998)
	*Rickettsia africae*	ESF‐5	GCA_000023005^a^	S	Rolain et al. (1998); Strand et al. (2017)
	*Rickettsia conorii*	A‐167	GCA_000261325^a^	S	Rolain et al. (1998)
		Malish 7	GCA_000007025^a^	S	Kim et al. (2019); Rolain et al. (1998)
		Malish 7 ‐ RIF. resistant	SRR8404402^b^	R*	Kim et al. (2019)
		ISTT CDC1	GCA_000263815^a^	S	Rolain et al. (1998)
		Moroccan	AF076435^c^	S	Rolain et al. (1998)
	*Rickettsia honei*	RB	GCA_000263055^a^	S	Rolain et al. (1998)
	*Rickettsia japonica*	YH	GCA_000283595^a^	S	Rolain et al. (1998)
	*Rickettsia massiliae*	MTU1	AF076433^c^	R	Rolain et al. (1998)
		AZT80	GCA_000283855^a^	R	Eremeeva et al. (2006)
		Bar‐29	AF076436^c^	R	Rolain et al. (1998)
	*Rickettsia montanensis*	VR‐611	n.a._	R	Rolain et al. (1998)
	*Rickettsia parkeri*	Maculatum 20	n.a.__	S	Rolain et al. (1998)
	*Rickettsia rhipicephali*	3‐7‐female6‐CWPP	GCA_000284075^a^	R	Rolain et al. (1998)
	*Rickettsia rickettsii*	R	GCA_000284075^a^	S	Rolain et al. (1998)
	*Rickettsia sibirica subsp. sibirica*	246	GCA_000166935^a^	S	Rolain et al. (1998)
	*Rickettsia sibirica subsp. mongolitimonae*	HA‐91	GCA_000247625^a^	S	Rolain et al. (1998)
	*Rickettsia slovaca*	13‐B	GCA_000237845^a^	S	Rolain et al. (1998)
Typhus	*Rickettsia prowazekii*	BreinI	GCA_000367405^a^	S	Miyamura et al. (1989); Rolain et al. (1998)
		Madrid E	GCA_000195735^a^	S	Rachek et al. (1998)
		Erif^r^1	n.a.__	R*	Rachek et al. (1998)
	*Rickettsia typhi*	Wilmington	GCA_000008045^a^	S	Rolain et al. (1998, 2002)
		Ethiopian ‐ RIF. resistant	n.a.__	R*	Troyer et al. (1998)
Transitional	*Rickettsia akari*	MK	n.a.__	S	Rolain et al. (1998)
	*Rickettsia australis*	Phillips	GCA_000273745^a^	S	Rolain et al. (1998)
	*Rickettsia felis*	URRWXCal2	GCA_000012145^a^	S	Rolain et al. (2002)
Helvetica	*Rickettsia helvetica*	C6P9	n.a.__	S	Rolain et al. (1998)
Canadensis	*Rickettsia canadensis*	2678	n.a.__	S	Rolain et al. (1998)
Bellii	*Rickettsia bellii*	369L42‐1	n.a.__	S	Rolain et al. (1998)

*Note*. R* indicates laboratory‐selected resistance through artificial mutagenesis or selection. GenBank accession numbers of *rpoB* gene sequences are indicated if available:

^a^ assembled genome sequences.

^b^ unassembled reads.

^c^ complete *rpoB* sequences; n.a., non‐available.

In bacteria, the most common mechanism underlying rifampin resistance involves missense mutations within the *rpoB* gene, which encodes the RNA polymerase β subunit (Goldstein, 2014; Koch et al., 2014; Tupin et al., 2010). In most bacterial genera, rifampin‐resistant clinical isolates typically harbor mutations that map to the center of the *rpoB* gene sequence in three clusters (I, II and III), at positions 500–700 corresponding to the enzyme's active center (Goldstein, 2014; Koch et al., 2014; Tupin et al., 2010). The majority of these mutations are located within a small region in cluster I dubbed the Rifampin Resistance Determining Region (RRDR). These mutations adversely impact the rifampin binding site, resulting in decreased affinity for the antibiotic (Goldstein, 2014; Koch et al., 2014; Tupin et al., 2010). Additionally, in the opportunistic pathogen *Nocardia farcinica*, the rifampin resistance mechanism also involves a *rpoB* paralog gene, which encodes a rifampin‐refractory β subunit (Ishikawa et al., 2006). In a few pathogenic bacteria, other alternative resistance mechanisms include rifampin inactivation by specific enzymes (Hoshino et al., 2009; Liu et al., 2018; Spanogiannopoulos et al., 2014; Stogios et al., 2016; Tribuddharat & Fennewald, 1999) or excretion by efflux systems, whereby bacteria pump out the antibiotics to the external environment using transporter proteins (Chandrasekaran & Lalithakumari, 1998; Hui et al., 1977; Louw et al., 2009).

In *Rickettsia*, rifampin resistance mechanisms have exclusively been associated with residue changes in the RNA polymerase β subunit, resulting from missense mutations in the *rpoB* gene (Drancourt & Raoult, 1999; Kim et al., 2019; Rachek et al., 1998; Troyer et al., 1998). Indeed, resistance associated with *rpoB* mutations has been artificially selected in the laboratory in three species that are primarily susceptible to rifampin, *R. conorii*, *R. typhi* and *R. prowazekii* (Kim et al., 2019; Rachek et al., 1998; Troyer et al., 1998) (Table 1). For naturally rifampin‐resistant species, a previous genetic investigation concluded that a single point *rpoB* mutation resulting in a phenylalanine‐to‐leucine change at position 973 (Phe‐973→Leu‐973) is the mechanism driving natural rifampin resistance (Drancourt & Raoult, 1999). However, this assertion was based on observations of *Rickettsia* species within the Spotted Fever group exclusively, and no further investigation has been conducted into other groups.

In this study, we investigate natural rifampin resistance patterns within the *Rickettsia* genus. We first assessed rifampin resistance in a previously untested *Rickettsia* species of the Transitional group, *R. lusitaniae*, for which no culture is currently available. To this aim, laboratory‐reared *Ornithodoros moubata* ticks naturally infected by the *R. lusitaniae* R‐Om strain (Duron et al., 2017, 2018) were subjected to rifampin treatment, and then *Rickettsia* density was monitored using specific qPCR assays. Subsequently, we compared the complete *rpoB* gene sequences of *R. lusitaniae* R‐Om with sequences of other *Rickettsia* species previously characterized as susceptible or resistant to rifampin and extended this analysis to include most other *Rickettsia* groups. We further explored available *Rickettsia* genomes for potential alternative resistance mechanisms, and retrace the evolutionary emergence of natural rifampin resistance in the genus. As a whole, our observations refute the prevailing notion that the residue Leu‐973 is the key driver of natural rifampin resistance in *Rickettsia* species.

## 2 EXPERIMENTAL PROCEDURES

### 2.1 Ticks, housing conditions and antibiotic treatment

Ticks were from a laboratory colony of *O. moubata* sensu stricto (Neuchâtel strain), which was established from field specimens collected in Southern Africa (Duron et al., 2018). Around two‐thirds of specimens of this laboratory colony are naturally infected with the *R. lusitaniae* R‐Om strain, which exhibits 100% nucleotide identity with the *gltA* gene sequence of the *R. lusitaniae* type strain (Duron et al., 2018) primarily identified in the tick *Ornithodoros erraticus* (Milhano et al., 2014).

Ticks were maintained in the laboratory at 26°C with 80–90% relative humidity under complete darkness (Buysse et al., 2021; Duron et al., 2018). A blood meal made of heparinized cow blood was offered to ticks every 7 weeks using an artificial feeding system. Ticks were allowed to feed on blood through a parafilm membrane using a specific apparatus including: (i) A tick chamber closed on top by a nylon cloth to avoid tick escape and closed below by the parafilm membrane, (ii) a blood chamber containing a magnet, and (iii) a hot magnetic steering device to mix and warm blood at 38°C. After feeding, each batch of ticks was kept in separate plastic containers until the next feeding.

To test for the antibiotic resistance pattern of *R. lusitaniae* R‐Om, a rifampin solution was added to the blood meal at a final concentration of 10 mg/ml (Duron et al., 2018). Twenty randomly sampled ticks were fed with rifampin‐treated blood, while 20 other ticks were fed with nontreated blood as a control. The ticks obtained their initial blood meal at nymphal stage 1, followed by another blood meal at nymphal stage 2 (7 weeks later). They were then kept until molting at nymphal stage 3, at which point they were analyzed to check the quantity of *Rickettsia*. No additional rifampin‐treated blood can be provided afterwards because, following two rifampin‐treated blood meals, most of the treated ticks ceased feeding. This was due to the antibiotic's elimination of their obligate nutritional endosymbiont, a *Francisella*‐like endosymbiont, required for their normal growth through B vitamin provisioning (Duron et al., 2018).

### 2.2 Fluorescence in situ hybridization and imaging

Visualization of *R. lusitaniae* was conducted through fluorescence in‐situ hybridization (FISH) assays following a protocol modified from Manz et al. (Manz et al., 1992). We focused on Malpighian tubes of ticks since these organs typically host a high density of intracellular bacteria (Buysse et al., 2019; Duron & Gottlieb, 2020). Malpighian tubules of adult *O. moubata* ticks were dissected and further fixed in 4% paraformaldehyde‐PBS‐0.1% Triton X‐100, for 1 h at room temperature. Thereafter, Malpighian tubules were washed twice in PBS for 15 min and stored at 4°C in 1:1 (v/v) ethanol‐PBS solution. The organs were dehydrated in 50%, 80% and 100% ethanol for 3 min each, then immersed in 50 µl of the following hybridization buffer: 900 mM NaCl, 35% formamide, 20 mM Tris‐HCl (pH 8), 0.01% SDS, 5 µL of the probe Rick_R2 (30 ng/µl, [CY3]‐TTTCTGCAAGTAACGTCATTATC, Eurofins). The probe is specific for *Rickettsia* spp. and was designed for this study. The organs were then hybridized for 1 h 30 min to 3 h maximum at 46°C, then washed for 20 min at 48°C in 200 µl of a washing buffer containing: 20 mM Tris (pH 8), 70 mM NaCl, 5 mM EDTA (pH 8) and 0.01% SDS 10%. Subsequently, the organs were gently rinsed in bi‐distilled water in a Petri dish placed on a glass slide, dried, and embedded in CitiDAPI (DAPI 10 mg.ml^−1^; Citifluor AF1 antifading, Citifluor, England). Negative controls were obtained by incubating organs without *Rickettsia*‐specific probes and by checking for tissue autofluorescence.

Confocal images were acquired on an Olympus confocal laser‐scanning microscope (Olympus IX81) and FV3000 2.0 software (Olympus), installed on an inverted microscope IX‐83 (Olympus, Tokyo, Japan). Multiple fluorescence images were acquired sequentially with a 60x objective (UplanXAPO, water immersion, 1.42 NA; Olympus) or a 100x objective (UPLAPO, oil immersion, 1.5 NA; Olympus). Fluorescence was excited with the Helium‐Neon laser line (543 nm, for Cy3) and a blue diode (405 nm, for DAPI) and the emitted fluorescence was detected through spectral detection channels between 430–470 and 570–670 nm, respectively.

### 2.3 Quantitative PCR assay

We developed a real‐time quantitative PCR assay (qPCR) to quantify the density of *R. lusitaniae* R‐Om strain in ticks. DNA was extracted from the tick's whole body using the DNeasy blood and tissue kit following the manufacturer's instructions (QIAGEN). qPCR was performed with a Light Cycler 480 (Roche) using the SYBR Green Master Mix. Two qPCRs were performed for each tick: one was specific for the *Rickettsia* R‐Om *gltA* gene, and the other was specific for the *O. moubata OmAct2* gene (Table A1 in Appendix 1). Since both genes are present in a single copy per haploid genome of the tick and the bacterium, the ratio between *gltA* and *OmAct2* concentrations provides the number of *Rickettsia* R‐Om genomes relative to the number of *O. moubata* genomes, thus correcting for the quality of DNA template. Each DNA template was analyzed in triplicate for *gltA* and *OmAct2* quantifications. Standard curves were plotted using dilutions of a pEX‐A2 vector (Eurofins) containing one copy of each of the *gltA* and *OmAct2* gene fragments.

### 2.4 Statistical analyses

All statistical analyses were carried out using R (https://www.r-project.org). We tested for the effect of rifampin treatment on *R. lusitaniae* through quantitative analyses. To determine if rifampin modifies the *R. lusitaniae* density within each infected tick, qPCR results of the control and treated groups were analyzed using a Wilcoxon‐Mann‐Whitney test.

### 2.5 Analysis of rpoB gene sequences

Neither genomic nor complete *rpoB* gene sequences of *R. lusitaniae* were available in public databases. Thus, we reexamined the raw reads from a prior metagenomics investigation of *O. moubata* Neuchâtel laboratory colony, specifically targeting the *Francisella*‐like endosymbiont also present in this tick species (Duron et al., 2018). The ticks used for the rifampin test were sourced from the same laboratory cohort, collected during the same period, and reared in identical conditions to those used for the metagenomics sequencing. Raw reads were mapped against the complete *rpoB* gene sequence of *R. felis* (GenBank CP000053, locus tag RF_1146) with BWA‐MEM (Li, 2013) and then retrieved to reconstruct the gene sequence using Megahit (v1.2.9) (Li et al., 2015).

We further compared the complete *rpoB* gene sequence of *R. lusitaniae* R‐Om strain obtained in this study with the complete *rpoB* gene sequences of *Rickettsia* spp. either susceptible (*n* = 16) or naturally resistant (*n* = 5) to rifampin available in GenBank (Table 1). Alignments of nucleotides and amino acids were performed using ClustalO (Sievers et al., 2011). The Unipro UGENE software (Okonechnikov et al., 2012) was used to visualize mutations throughout the *rpoB* gene sequences, and the BioEdit software (Hall, 1999) to identify residues with analogous functions in proteins. The resistance clusters I, II, and III were identified along the *Rickettsia rpoB* gene sequences through alignment with the *rpoB* sequence of *E. coli* strain NCM3722 (GenBank CP011495; clusters I, II, and III at positions 507–533, 563–572, and 678, respectively). The final alignments of the *rpoB* gene sequences were then used to identify mutations specifically associated with natural rifampin resistance in the genus *Rickettsia*. Consensus sequence logos were created from aligned *rpoB* gene sequences of rifampin‐resistant and susceptible *Rickettsia* species and strains (Gagniuc, 2021). We also examined all *Rickettsia* genomes for the presence of *rpoB* paralogs using tBLASTn (Altschul et al., 1990).

We further expanded the analysis of *rpoB* gene sequences to a global set of representative *Rickettsia* species and strains with genomic sequences available on public databases, including strains whose rifampin resistance phenotype is unknown (Table A2 in Appendix 1). The whole genomes of the *Rickettsia* species and strains were then used for phylogenomics. The *Rickettsia* genomes were processed with a standardized genome annotation approach using Prokka (Seemann, 2014), and single‐copy orthologs (SCOs) were next identified using OrthoFinder (v2.3.11) (Emms & Kelly, 2019). SCOs were individually aligned with MAFFT (v7.450) (Katoh & Standley, 2013) and ambiguous positions were removed using trimAl (v1.2rev59) (Capella‐Gutiérrez et al., 2009) before individual alignments concatenation using Amas 1.0 (Borowiec, 2016). The best substitution models were determined using modeltest (v0.1.5) (Darriba et al., 2020) and maximum likelihood (ML) trees were computed using RAxML (v8.2.9) (Stamatakis, 2014) with 1,000 bootstrap replicates.

**Table 2:** List of candidate genes potentially associated with natural rifampin resistance.

Gene (locus tag^a^)	Putative gene product	Length (amino acids)
Genes present in all *Rickettsia* genomes but harboring residues specific to rifampin‐resistant species		
*rpoB* (MCC_01550^a^)	β subunit of RNA polymerase	1373
*rpoA* (MCC_06070^a^)	α subunit of RNA polymerase	340
*rpoC* (MCC_01555^a^)	β‘ subunit of RNA polymerase	1372
*rpoD* (MCC_00410^a^)	σ subunit of RNA polymerase	634
*rpoZ* (MCC_05500^a^)	ω subunit of RNA polymerase	127
*YajC* (MCC_05555^a^)	Subunit of the Sec membrane complex	141
*TolC* (MCC_02285^a^)	Outer membrane porin	453
*MsbA1* (*efrA*) (MCC_03565^a^)	Subunit of a multidrug efflux ABC transporter	459
Genes only present in rifampin‐resistant *Rickettsia* species (without orthologs in susceptible species)		
Rrhi37F6_00877^b^	Hypothetical protein	192
Rrhi37F6_00879^b^	Hypothetical protein	62
Rrhi37F6_01547^b^ *(CopG)*	Helix‐turn‐helix protein	76
Rrhi37F6_01549^b^	Predicted transcriptional regulators containing the CopG/Arc/MetJ DNA‐binding domain and a metal‐binding domain	59
Rrhi37F6_00531^b^ *(ompA)*	Outer membrane protein (truncated)	97
Rrhi37F6_01548^b^ *(ParA*)	Plasmid stability protein	213
Rrhi37F6_00259^b^	Hypothetical protein	68
Rrhi37F6_00257^b^	IS982 family transposase (pseudogenized)	65
Rrhi37F6_01535^b^	Transposase (pseudogenized)	37
Rrhi37F6_01536^b^	Guanosine polyphosphate pyrophosphohydrolase/synthetase	937
Rrhi37F6_01542^b^	IS3 family transposase (pseudogenized)	55

^a^ Locus tags in the genome of *Rickettsia rhipicephali* strain 3‐7‐female6‐CWPP (GenBank CP003342.1).

^b^ Putative additional genes without orthologs in rifampin‐susceptible *Rickettsia* species, identified in this study, in the genome of *R. rhipicephali* strain 3‐7‐female6‐CWPP. Details are presented in Table A3 and Figure A1 in Appendix 1.

The 3D structures of the beta subunit of two rifampin‐sensitive species (*R. conorii* A‐167 and *R. rickettsii* R) and two rifampin‐resistant (*R. rhipicephali* 3‐7‐female‐6‐CWPP and *R. aeschlimannii* MC16) were modeled using Colab AlphaFold2 (Jumper et al., 2021; Mirdita et al., 2022). PrankWeb 3 (https://prankweb.cz/) was then used to predict potential ligand‐binding sites for each subunit (Jakubec et al., 2022). The predicted sites were compared to the rifampin/beta subunit attachment site from the *Mycobacterium tuberculosis* crystal structure (accession 5UHB in the RCSB Protein Data Bank; https://www.rcsb.org/) to identify the hypothetical rifampin‐binding sites on the beta subunits of *R. conorii* A‐167, *R. rickettsii* R, *R. rhipicephali* 3‐7‐female‐6‐CWPP and *R. aeschlimannii* MC16.

### 2.6 Alternative rifampin resistance mechanisms in Rickettsia genomes

We further investigated the whole genomes of *Rickettsia* for potential alternative mechanisms associated with natural rifampin resistance that are not dependent on *rpoB*. We compared the whole genomic content of naturally rifampin‐resistant (*n* = 3) and ‐susceptible (*n* = 15) *Rickettsia* species (Table A2 in Appendix 1) to identify orthogroups specific to the rifampin‐resistant *Rickettsia* species. The list of orthogroups specific to naturally rifampin‐resistant *Rickettsia* species were then obtained using Orthofinder (v2.3.11) (Emms & Kelly, 2019) and Roary (Page et al., 2015). To control for false positives, we used tBLASTn to verify that orthogroups putatively associated with natural rifampin resistance were indeed absent in rifampin‐susceptible species.

We used tBLASTn to check whole genomes of naturally rifampin‐resistant (*n* = 3) or susceptible *Rickettsia* species (*n* = 15) for the presence of genes known to encode enzymes inactivating rifampin and efflux systems pumping out rifampin described in rifampin‐resistant bacteria (Brandis et al., 2012; Comas et al., 2011; Song et al., 2014). In addition, we used the Resistance Gene Identifier (RGI) tool of the Comprehensive Antibiotic Resistance Database (CARD) (Jia et al., 2017). The RGI tool was parametrized to search for genes associated with rifamycin resistance (the class of antibiotics including rifampin) and to generate a list of putative rifamycin resistance genes in *Rickettsia* genomes.

## 3 RESULTS

### 3.1 Rickettsia lusitaniae R‐Om is susceptible to rifampin

**Figure 1:**
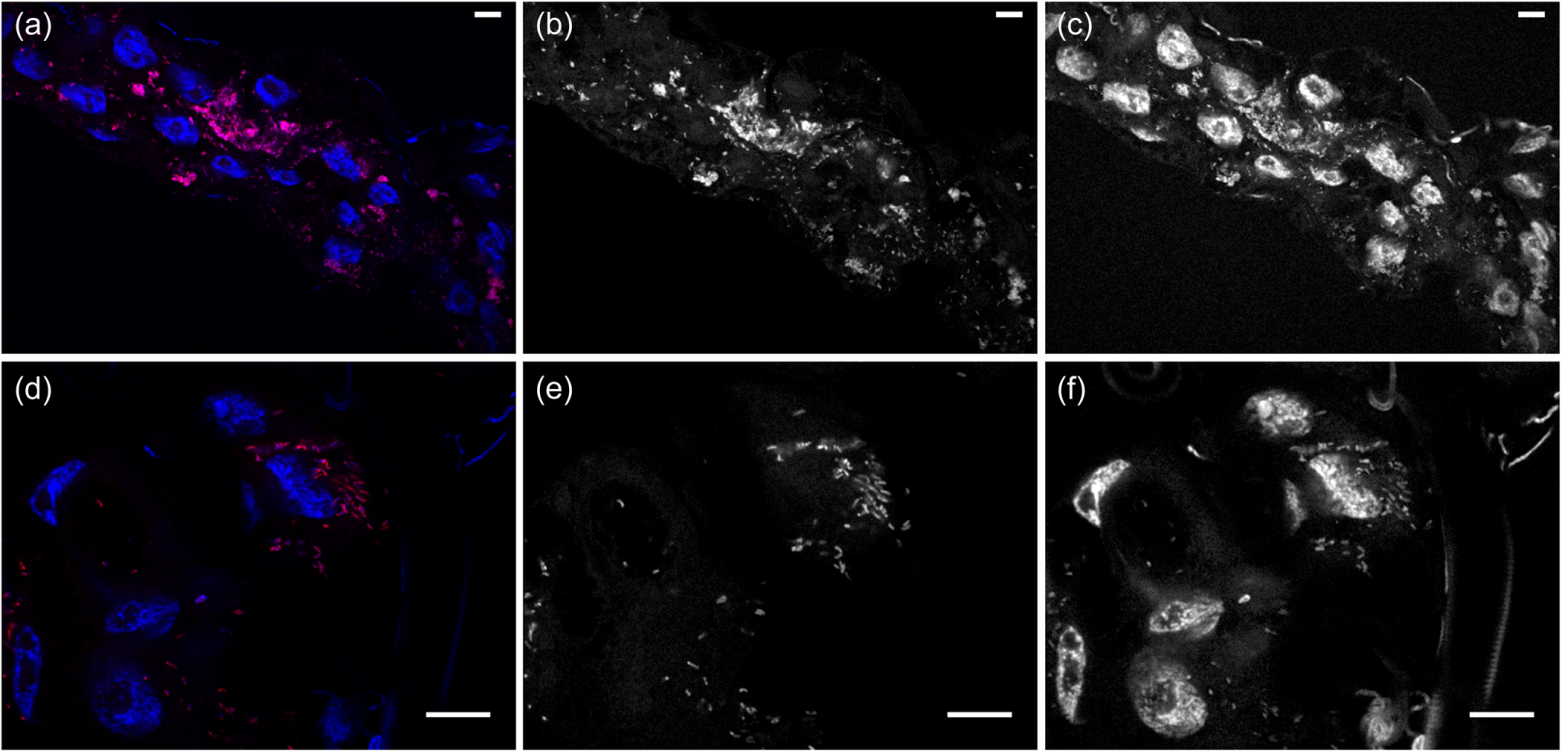
Detection of *Rickettsia lusitaniae* R‐Om using epifluorescent microscopy within Malpighian tubules of the tick *O. moubata*. (a, d) *R. lusitaniae* R‐Om appears in purple owing to the co‐localization between the FISH probe in red (greyscale: b, e) and the DAPI staining in blue (greyscale: c, f). The nuclei are labeled in blue with DAPI only (greyscale: c, f). *Rickettsia lusitaniae* R‐Om tends to cluster nearby nuclei and are rod‐shaped. Scale bars: 10 µm.

**Figure 2:**
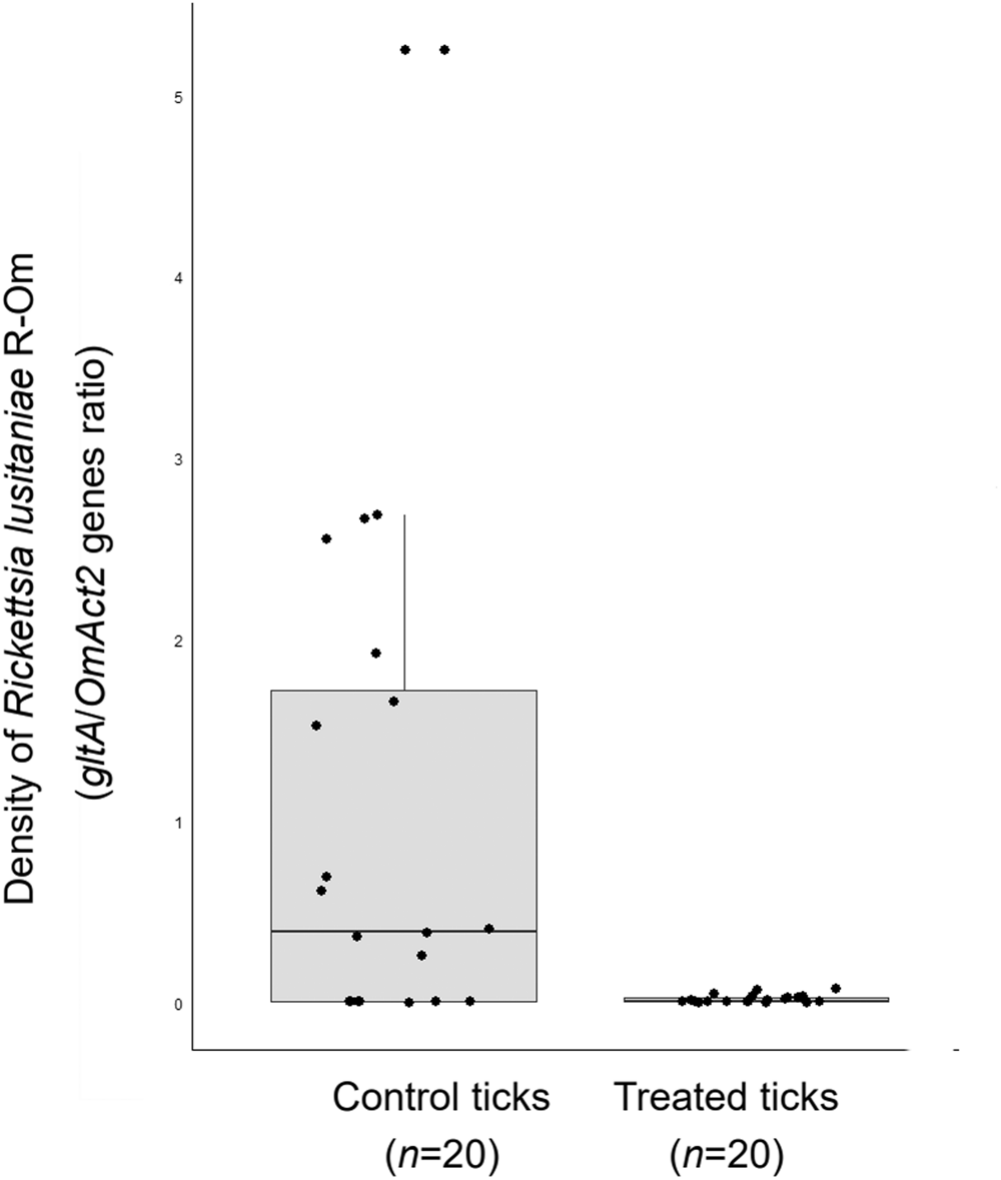
Effect of rifampin treatment on the density of *Rickettsia lusitaniae* R‐Om in third instar nymphs of the tick *Ornithodoros moubata*. Boxplots show *Rickettsia lusitaniae* R‐Om densities in control and rifampin‐treated groups. Infection densities in ticks were quantified through qPCR by the ratio of *Rickettsia* R‐Om *gltA* gene per *O. moubata OmAct2* gene.

FISH confirmed the presence of *R. lusitaniae* R‐Om in *O. moubata* and revealed a high concentration of *Rickettsia* in Malpighian tubules (Figure 1a–f). Real‐time qPCR also showed that *R. lusitaniae* R‐Om is present at high concentration in most ticks fed on untreated blood (control ticks) (Figure 2a,b). A bimodal distribution is observed for the controls, with seven out of 20 ticks having an estimated *R. lusitaniae* R‐Om concentration close to 0. This infection pattern was expected since not all *O. moubata* specimens in this lab colony are infected by *R. lusitaniae* R‐Om (Duron et al., 2018). However, the density of *R. lusitaniae* R‐Om was 58x lower in the rifampin‐treated group (average of *gltA*/*OmAct2* ratios ± SE: 0.018 ± 0.020, *n* = 20) than in the control group (mean ± SE: 1.048 ± 1.386, *n* = 20) (Wilcoxon‐Mann‐Whitney test, two‐sided, *p* = 0.002). This result indicates that *R. lusitaniae* R‐Om is susceptible to rifampin.

### 3.2 Inaccuracy of the rpoB Phe‐973→Leu‐973 mutation for rifampin resistance

**Figure 3:**
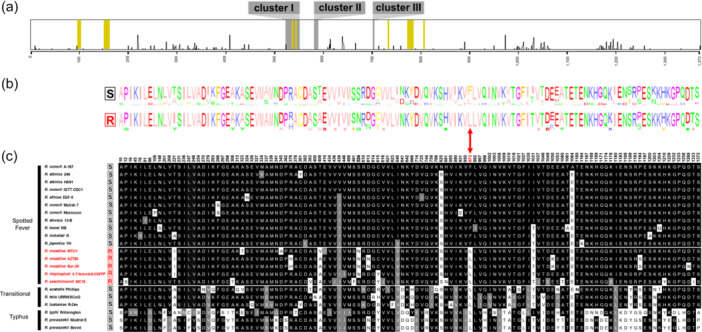
Alignment of 1373 amino acids of the *rpoB* gene for *Rickettsia* strains and species either susceptible or resistant to rifampin. (a) Highlights of residues variable along the *rpoB* gene sequence. The height of each peak indicates the proportion of *Rickettsia* strains and species harboring amino acid residues variable along the sequence. Only one polymorphic residue is located in one of the three clusters I, II, and III. The residues in contact with the hypothetical rifampicin binding site are annotated in yellow. (b) Consensus sequence logos of amino acid variants in the *rpoB* gene sequences for rifampin‐resistant (R) and susceptible (S) *Rickettsia* species and strains. (c) Details of variable residues in the *rpoB* gene sequences of the *Rickettsia* strains and species. Positions with conserved residues compared to *R. conorii* A‐167 placed as reference are depicted in black; positions with substitutions by analogous residues are shown in gray; positions with substitutions by non‐analogous residues are represented in white. The red arrow points to the residues Leu‐973 and Phe‐973.

Alignments of *rpoB* gene sequences showed that the mutation Phe‐973→Leu‐973 primarily associated with natural rifampin resistance do not explain alone the pattern observed in *Rickettsia* species and strains. As expected, the residue Leu‐973 is present in naturally rifampin‐resistant *Rickettsia* species and strains and absent in susceptible species and strains of the Spotted Fever group (Figure 3a–c). However, the residue Leu‐973 is also present in all rifampin‐susceptible *Rickettsia* species belonging to the other *Rickettsia* groups (Figures 3b,c). Indeed, analysis of raw reads obtained from the *O. moubata* metagenome allowed us to reconstruct the complete *rpoB* gene sequence of the *R. lusitaniae* R‐Om strain (length: 4123 nucleotides; 1373 amino acids). While the *R. lusitaniae* R‐Om strain is susceptible to rifampin, the residue Leu‐973 is present in its *rpoB* gene sequence. Similarly, the residue Leu‐973 is consistently present in other rifampin‐susceptible *Rickettsia* species of the Transitional group (*R. australis*, *R. felis*) and in rifampin‐susceptible *Rickettsia* species of the Typhus group (*R. typhi*, and *R. prowazekii*) (Figures 3b,c). As a result, the residue Leu‐973 is not specific to naturally rifampin‐resistant *Rickettsia* species.

**Figure 4:**
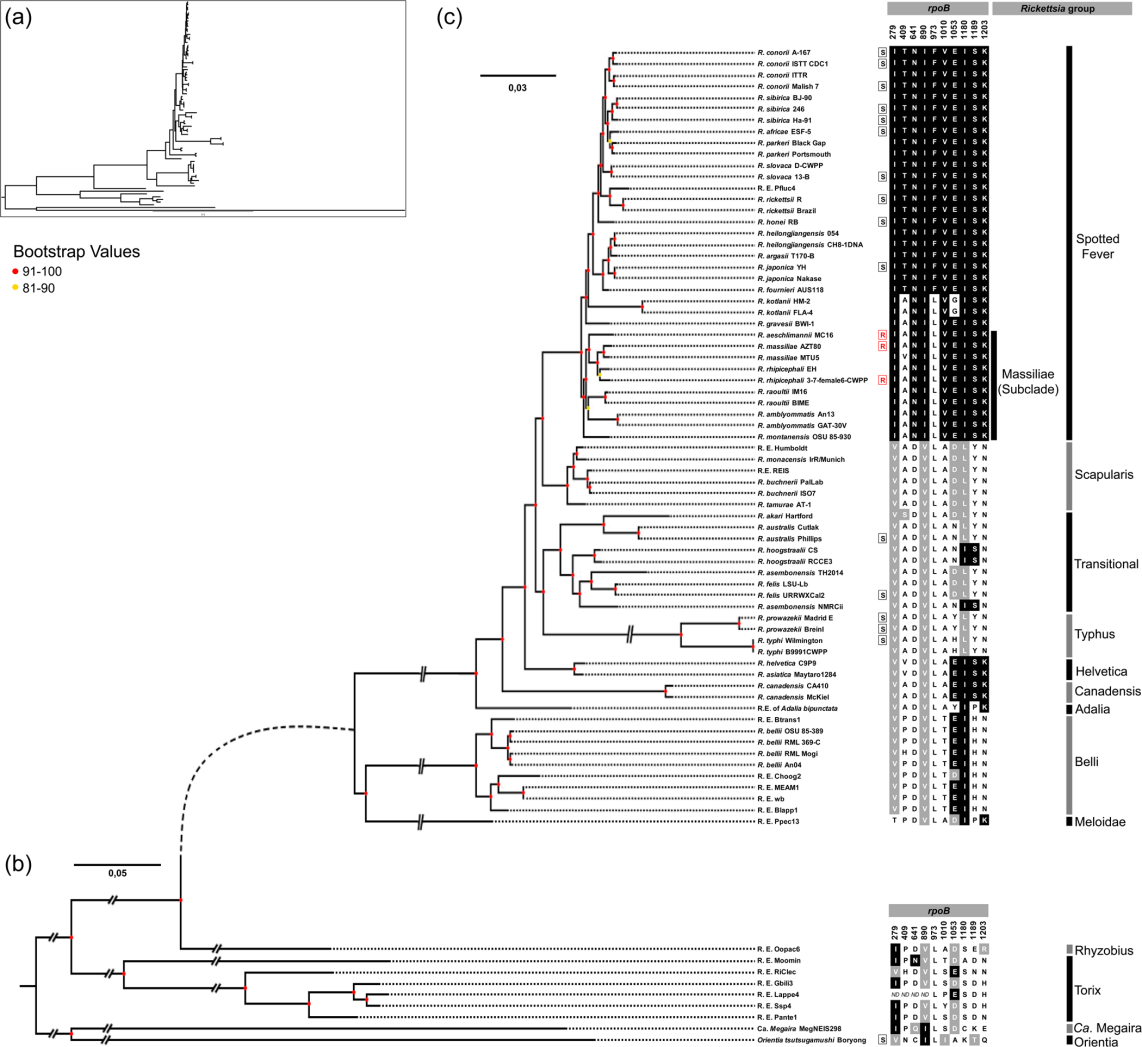
Whole‐genome phylogenetic relationship in the genus *Rickettsia*. (a) Whole‐genome phylogenetic relationship of the *Rickettsia* species and strains (including the two outgroups *Candidatus Megaira* strain MegaNEIS298 and *Orientia tsutsugamushi* strain Boryong) constructed from 229 SCOs (56,554 amino acids) (ML, JTT + I + G4 model). (b) Focus on the whole‐genome phylogenetic relationship of species of the Rhyzobius and Torix groups, including outgroups (*Orientia* and Ca. *Megaira* species). Analysis was based on *Rickettsia* species and strains from 229 SCOs (56,554 amino acids) (ML, JTT + I + G4 model). (c) Focus on the whole‐genome phylogenetic relationship of species of the Spotted Fever, Scapularis, Transitional, Typhus, Helvetica, Canadensis, Adalia and Belli groups. Analysis was constructed from 402 SCOs (99,416 amino acids) (ML, FLU + I + G4 model). R (in red), naturally rifampin‐resistant *Rickettsia* species and strains; S (in black), rifampin‐susceptible *Rickettsia* species and strains. The 10 residues associated with rifampin resistance are shown on the right of the trees. Conserved amino acid residues are represented in black across all or diverse sequences. Positions with conserved residues are depicted in black; positions with substitutions by analogous residues are shown in gray; positions with substitutions by non‐analogous residues are represented in white. Clade robustness was assessed by bootstrap analysis using 1000 replicates.

Further comparisons with the *rpoB* gene sequences of additional *Rickettsia* strains and species for which the rifampin resistance profile is unknown showed that the residue Leu‐973 is conserved across all *Rickettsia* groups (Figure 4a–c). Only the rifampin‐susceptible species belonging to the Spotted Fever group do not harbor the residue Leu‐973, but instead harbor Phe‐973. Examination of *Rickettsia* genomes confirmed that *rpoB* is a single‐copy gene, with no paralog present in the genus. Phylogenomic analyses revealed that the residue Leu‐973 is ancestral to the *Rickettsia* genus. The residue Phe‐973 is rather a derived trait, which has only evolved in a subclade of the Spotted Fever group from the ancestral residue Leu‐973 (Figure 4a–c). In this context, the nomenclature Leu‐973→Phe‐973 is thus more appropriate.

Phylogenomic analyses also indicated that all naturally rifampin‐resistant species cluster in a monophyletic subclade, termed Massiliae, of the Spotted Fever group (Figure 4c). Remarkably, the Massiliae subclade also includes a number of *Rickettsia* strains and species, for which the rifampin resistance pattern is unknown: *R. massiliae* (strain MTU5), *R. rhipicephali* (EH), *R. montanensis* (OSU 85‐930), *R. raoultii* (BIME, IM16), and *R. amblyommatis* (An13, GAT‐30V).

### 3.3 Distinct sets of mutations in rpoB are associated with naturally rifampin‐resistant Rickettsia

**Figure 5:**
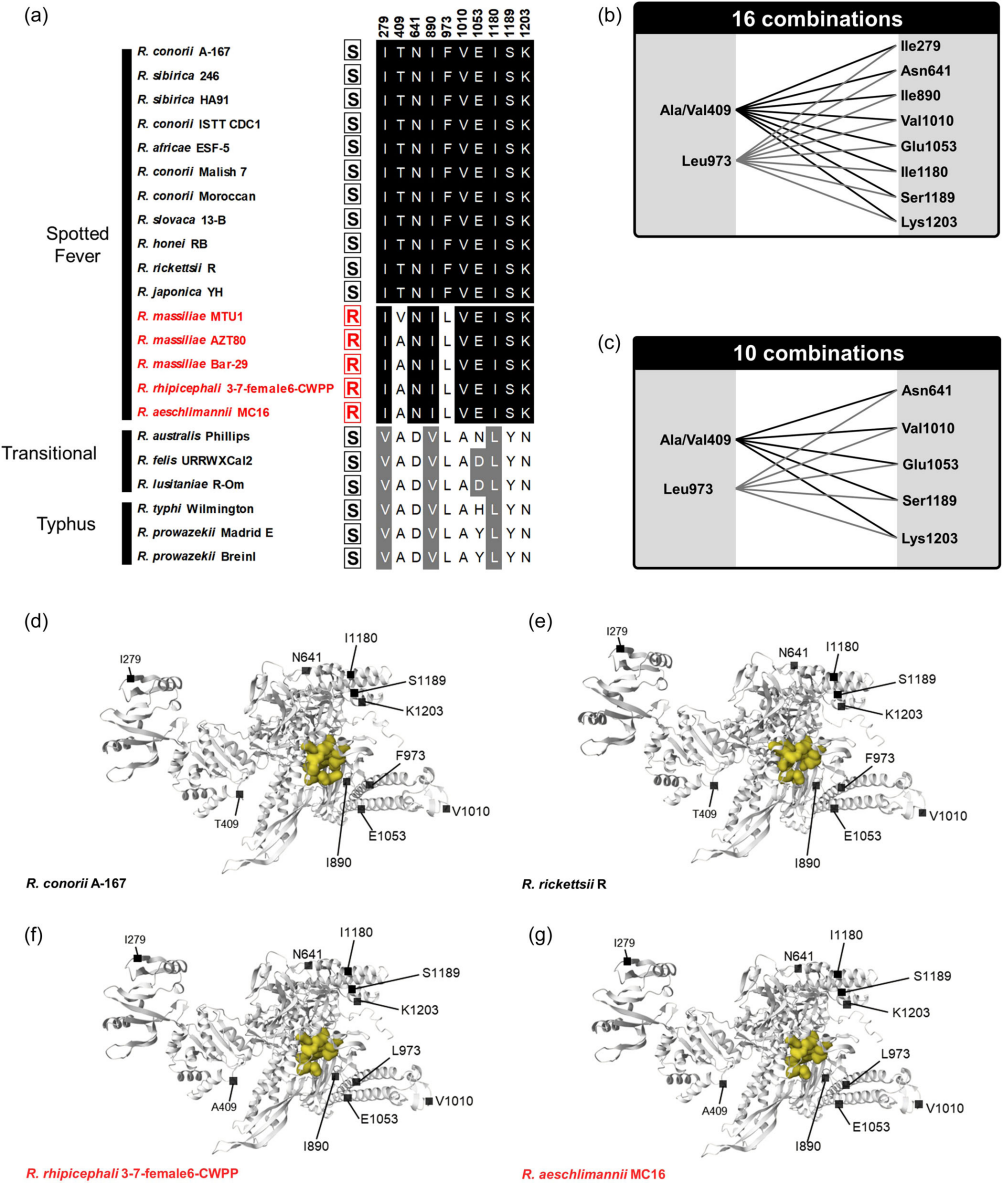
Residues in the *rpoB* gene sequences specific to rifampin‐resistant and susceptible *Rickettsia* species and strains. (a) The 10 residue putatively associated with rifampin resistance. (b) The 16 residue pairing (including substitutions by analogous and non‐analogous residues) associated with rifampin resistance. (c) The 10 residue pairings (including only non‐analogous residues) associated with rifampin resistance. R (in red), naturally rifampin‐resistant *Rickettsia* species and strains; S (in black), rifampin‐susceptible *Rickettsia* species and strains. (d) 3D structure of the beta subunit of *R. conorii* A‐167 (rifampin‐sensitive), (e) *R. rickettsi* R (rifampin‐sensitive), (f) *R. rhipicephali* 3‐7‐female‐6‐CWPP (rifampin‐resistant), and (g) *R. aeschlimannii* MC16 (rifampin‐resistant). Candidate residues for rifampin sensitivity/resistance are shown. The hypothetical rifampin binding site is indicated in yellow.

The rifampin‐resistant clusters I, II, and III exhibited a low amino acid polymorphism in the genus *Rickettsia*, with only one amino acid variant identified in cluster I in RRDR (Ser‐524→Asn‐524, Figure 3a). The residue Asn‐524 is associated with some naturally rifampin‐resistant species, but not all: *R. aeschlimani* strain MC16 harbors the residue Ser‐524, which is shared with all the susceptible species, suggesting that residue Asn‐524 is not involved in natural rifampin resistance. Outside of the three cluster regions, there is no single specific residue in the *rpoB* sequences associated with naturally rifampin‐resistant species (Figures 3b,c). Notably, we identified a set of 10 residues (Ile‐279, Ala/Val‐409, Asn‐641, Ile‐890, Leu‐973, Val‐1010, Glu‐1053, Ile‐1180, Ser‐1189, and Lys‐1203) which are shared by naturally rifampin‐resistant species, but also with part of susceptible species (Figure 5a). However, pairing specific residues at positions 409 or 973 with specific residues at positions 279, 641, 890, 1010, 1053, 1180, 1189, or 1203 fits the rifampin resistance pattern (Figures 5a,b). Indeed, the pairing of Leu‐973 and Ser‐1189 is specific to rifampin‐resistant *Rickettsia* and is never found in other *Rickettsia* species and strains. A total of 16 potential pairings exist, each matching the resistance pattern (Figures 5a,b). None of these residues is located within the hypothetical rifampin binding site (Figure 5d–g). Although there are a few subtle variations between *Rickettsia* species, no noticeable differences are observed between the hypothetical rifampin binding sites of resistant and sensitive *Rickettsia* species (Figure 5d–g). However, eight of these 10 residues (Ile‐279, Ala/Val‐409, Asn‐641, Ile‐890, Val‐1010, Glu‐1053, Ile‐1180, and Lys‐1203) have larger or similar side chains compared to the residues present in rifampin‐susceptible species. Only two residues (Leu‐973 and Ser‐1189) have smaller or similar side chains to those observed in rifampin‐susceptible species. Hence, the RNA polymerase β subunit of naturally rifampin‐resistant species tends to contain a higher proportion of residues with larger side chains than those of susceptible species. The steric hindrance may potentially reduce its susceptibility to binding with rifampin.

Furthermore, the number of residue pairs putatively associated with resistance patterns can be reduced by considering exclusively non‐analogous residues (i.e., amino acids with different chemical or functional properties) differing between rifampin‐resistant and susceptible species. This approach led to the exclusion of residues 279, 890, and 1180 (Figure 5c). Indeed, at each of these three positions, residues in resistant and susceptible species belong to the aliphatic hydrophobic group and share similar chemical properties. Hence, this could limit the number of residue pairing putatively associated with rifampin resistance to 10, all of which were absent in susceptible species (Figure 5c).

All the residue combinations putatively associated with natural rifampin resistance are also present in members of the Massiliae subclade. This also includes species and strains for which resistance pattern has never been tested: *R. massiliae* (strain MUT5), *R. rhipicephali* (EH), *R. montanensis* (OSU 85‐930), *R. raoultii* (BIME, IM16), and *R. amblyommatis* (An13, GAT‐30V) (Figure 4c). In addition, two species of the Spotted Fever Group, *R. gravesii* (BWI‐1) and *R. kotlanii* (HM‐2, FLA‐4), harbor the same (or almost) residue combinations, although they do not belong to the Massiliae subclade (Figure 4c). No other species share these combinations of residues.

### 3.4 Putative other mechanisms of rifampin resistance

**Figure A1:**
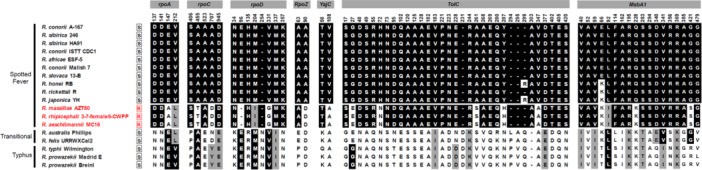
Missense mutations identified in the gene sequences of *rpoA*, *rpoC*, *rpoD*, *rpoZ*, *YajC*, *TolC*, and *MsbA1* specific to rifampicin‐resistant and susceptible *Rickettsia* species and strains. R (in red) denotes naturally rifampicin‐resistant *Rickettsia* species and strains, while S (in black) represents rifampicin‐susceptible *Rickettsia* species and strains. Positions with conserved residues are depicted in black; positions with substitutions by analogous residues are shown in gray; positions with substitutions by non‐analogous residues are represented in white; positions with deletions are shown by hyphens.

Examination of *Rickettsia* genomes reveals the presence of 18 additional genes potentially involved in natural rifampin resistance (Table 2, Table A3 and Figure A1 in Appendix 1). These candidate genes include eight genes potentially associated with rifampin metabolism and harboring specific mutation polymorphisms, and 11 other genes present in naturally resistant *Rickettsia* species, but absent in susceptible species. The *Rickettsia* genomes contain no homologs for genes encoding enzymes involved in rifampin inactivation present in *N. farcinica* (Hoshino et al., 2009; Liu et al., 2018), *Pseudomonas aeruginosa* (Tribuddharat & Fennewald, 1999) and *Listeria monocytogenes* (Spanogiannopoulos et al., 2014; Stogios et al., 2016).

Five candidate genes are involved with *rpoB* in the formation of the RNA polymerase complex: *rpoA* (α subunits), *rpoC* (β’ subunit), *rpoD* (σ subunit), and *rpoZ* (ω subunit) genes (Table 2). Three of them (*rpoA*, *rpoC* and *rpoD*) harbor at least one residue specific to rifampin‐resistant strains (Figure A1 in Appendix 1). Although these RNA polymerase subunits are not the binding site of rifampin, they are physically organized all around the β subunit. Three other candidate genes are involved in antibiotic efflux pump systems, consistently present in all *Rickettsia* genomes, and also harbor residue specific to rifampin‐resistant strains: *YajC* (encoding a subunit of the Sec membrane complex), *TolC* (a porin of outer membrane), and *MsbA1* (a subunit of a multidrug efflux ABC transporter) (Table 2, Figure A1 in Appendix 1).

All the 11 other candidate genes have been identified through pangenomic analyses and found specifically present in naturally rifampin‐resistant *Rickettsia* species and absent in susceptible species (Table 2, Table A3 in Appendix 1). These 11 candidate genes are present either in the main chromosome (*n* = 5) or in plasmids (*n* = 6) of naturally rifampin‐resistant *Rickettsia* species. However, none of these 11 genes could be associated with antibiotic resistance. One gene is homolog to *ParA*, which encodes a plasmid stability protein driving the isolation and allocation of plasmids into daughter cells during cell division (Ebersbach & Gerdes, 2005). Another gene is homolog to *CopG*, which encodes a DNA‐binding protein involved in the control of plasmid copy number (Gomis‐Ruth, 1998). The nine other candidate genes encode for short truncated protein fragments: One for a 97 amino acid fragment of the surface antigen encoded by the *ompA* gene, three for pseudogenized transposases, and five for short hypothetical proteins (59–92 amino acids) of unknown functions (Table 2, Table A3 in Appendix 1).

## 4 DISCUSSION

Our analysis of current genetic data expands our understanding of the mechanisms and evolution of rifampin resistance within the *Rickettsia genus*. Indeed, we characterize *R. lusitaniae* as a species susceptible to rifampin although its *rpoB* gene sequence contains the residue Leu‐973. We further observe that the residue Leu‐973 is conserved in all *Rickettsia* groups, being present in all rifampin‐susceptible species except those of the Spotted Fever group. Consequently, the *rpoB* residue Leu‐973 solely cannot be further used to diagnose natural rifampin resistance for *Rickettsia* species and strains. Alternative resistance mechanisms thus exist, which could involve either mutations reducing access to the rifampin binding site through alterations in the structure of the RNA polymerase β subunit or related subunits, or genes detoxifying rifampin through efflux pumps. There is no genomic evidence suggesting that *Rickettsia* can inactivate rifampin by a specific enzymatic activity. Crucially, the observed resistance pattern across *Rickettsia* groups shows that natural rifampin resistance is restricted to a unique monophyletic subclade, and potentially to a few other related species, within the Spotted Fever group. This pattern reveals that the emergence of natural rifampin resistance was driven by a major phylogenetic constraint, resulting from ancient genomic features shared by a unique set of closely related *Rickettsia* species, rather than being a consequence of recent selection due to exposure to rifampin. Such a phylogenetic constraint implies that the mutations specific to rifampin‐resistant *Rickettsia* species may be conserved due to shared evolutionary history rather than being directly related to rifampin resistance. Consequently, this phylogenetic constraint obscures the true genetic factors responsible for rifampin resistance in the genus *Rickettsia*.

The rifampin resistance mechanism of *Rickettsia* is distinct from mechanisms observed in most other resistant bacteria. While an accumulation of missense mutations in RRDR is typically observed in rifampin‐resistant bacteria (Forrest & Tamura, 2010; Goldstein, 2014), this pattern is not observed in RRDR of naturally resistant *Rickettsia* species. Polymorphism of residues exists in other *rpoB* regions, and some residues, if combined two‐by‐two, perfectly match the natural rifampin resistance phenotype. These mutations induce no substantial structural changes in the RNA polymerase β subunit, but they most often code for amino acids with larger side chains than those found in susceptible *Rickettsia* species. This can result in crowding at the binding site, thereby inhibiting the rifampin molecules from binding to the RNA polymerase. Similarly, the α, β’, and σ subunits, all assembled in close proximity to the β subunit in the RNA polymerase, harbor missense mutations specific to naturally rifampin‐resistant species. These peripheral structural changes can also prevent or limit rifampin access to the binding site on the RNA polymerase β subunit and then confer resistance to *Rickettsia* species, as suggested in a few other bacteria (Brandis et al., 2012; Comas et al., 2011; Liu et al., 2018; Song et al., 2014). However, while changes in the β subunit typically result in high‐level rifampin resistance, alterations in other RNA polymerase subunits lead to smaller, yet still substantial, reductions in susceptibility to rifampin (Brandis et al., 2012).

Efflux pumps are present in both rifampin‐resistant and susceptible *Rickettsia* species, but three of their key genes, *YajC*, *TolC*, and *MsbA1*, harbor residues specific to naturally resistant species. The *YajC* and *TolC* genes are involved in the regulation of the main multidrug efflux machinery, imparting resistance to broad‐spectrum antibiotics in bacteria (Du et al., 2015; Gill & Garcia, 2011; Jia et al., 2022; Okusu et al., 1996; Ramos et al., 2014). *MsbA1* is a drug transporter gene that forms part of the inner membrane ATP‐binding cassette (ABC) transporter, which can extrude antibiotics from the cell and induce resistance (Alexander et al., 2018; Díez‐Aguilar et al., 2021; Jia et al., 2022; Reuter et al., 2003; Woebking et al., 2008). In addition, analysis of the pangenome of naturally rifampin‐resistant *Rickettsia* species led to the identification of a specific duplication of the porin *ompA* gene. In bacteria, *ompA* plays a crucial role in regulating cellular permeability and can be associated with efflux systems in the inner membrane to facilitate the extrusion of antibiotics (Choi & Lee, 2019; Nie et al., 2020). However, the second copy of the *ompA* gene in naturally rifampin‐resistant *Rickettsia* species is truncated and may be nonfunctional, hindering any definitive conclusions. Additional analyses are required to validate the role of these alternative mechanisms of natural rifampin resistance in *Rickettsia* species.

Analysis of *Rickettsia* phylogeny reveals that the distribution of natural rifampin resistance is not random across species and suffers from a major phylogenetic constraint. Rifampin‐susceptible species are scattered along the phylogeny, belonging to different groups, suggesting that rifampin‐susceptibility is an ancestral trait in the genus *Rickettsia*. Furthermore, the extended genome‐wide analysis also revealed that some other *Rickettsia* species of the Spotted Fever group, non‐tested for rifampin resistance, shared key genetic features with species known to be naturally rifampicin‐resistant. These non‐tested species include *R. amblyommatis* and *R. raoultii*, which also belong to the Massiliae subclade, suggesting that they may be other naturally rifampin‐resistant species. In addition, two other non‐tested species, *R. gravesii* and *R. kotlanii*, shared similar genetic features with species known to be naturally rifampicin‐resistant, including key residues in their RNA polymerase β subunit, suggesting that they could be other resistant species. However, neither *R. gravesii* nor *R. kotlanii* belong to the Massiliae subclade, although they belong to the Spotted Fever group. All susceptible species in the Spotted fever group cluster in a distinct monophyletic subclade nested among the resistant or putative resistant species, suggesting that they have a unique evolutionary origin. The emergence of rifampin resistance is found at the root of the Spotted Fever group, as indicated by the phylogenetic partition of resistant (or putative resistant) versus susceptible strains. Additionally, resistance has possibly reverted to susceptibility during the subsequent diversification of Spotted Fever species. Previous phylogenetic investigations have estimated the origin of the Spotted Fever group to be 25 million years ago (Weinert et al., 2009; Weinert, 2015), providing unequivocal evidence that the emergence of natural rifampin resistance predates the use of antibiotics by humans.

To conclude, our study challenges previous assumptions regarding natural rifampin resistance in *Rickettsia*. Currently, the most reliable predictor of natural rifampin resistance is based on phylogenetic pattern: This phenotype is inherently linked with the Massiliae subclade, although it is likely that other closely related species, such as *R. gravesii* and *R. kotlanii*, are also resistant. These observations emphasize the importance of ongoing surveillance and research to understand how rifampin interacts with rickettsial targets and how *Rickettsia* can evolve antibiotic resistance. This is particularly crucial considering that new *Rickettsia* strains, species and groups are described each year, frequently without information on their antibiotic resistance status (Binetruy et al., 2020; Buysse & Duron, 2020; Hajduskova et al., 2016; Lacroux et al., 2023; Weinert et al., 2009).

## AUTHOR CONTRIBUTIONS

**Julien Amoros**: Data curation; Formal analysis; Visualization; Writing—original draft; Methodology; Investigation; Writing—review and editing. **Noor Fattar**: Data curation; Formal analysis; Visualization; Writing—original draft; Methodology; Investigation; Writing—review and editing. **Marie Buysse**: Data curation; Formal analysis; Visualization; Writing ‐ original draft; Methodology; Investigation; Writing— review and editing. **Meriem Louni**: Data curation; Formal analysis; Visualization; Writing—original draft; Methodology; Investigation; Writing—review and editing. **Joanne Bertaux**: Formal analysis; Visualization; Writing—original draft; Methodology; Investigation; Writing—review and editing; Data curation. **Didier Bouchon**: Conceptualization; Writing—original draft; Writing—review and editing; Project administration. **Olivier Duron**: Conceptualization; Writing—original draft; Writing—review and editing; Project administration; Supervision; Funding acquisition; Resources; Investigation; Validation.

## CONFLICT OF INTEREST STATEMENT

None declared.

## ETHICS STATEMENT

Tick feeding and manipulation were performed in a Biosafety Level 2 insectarium (Baillarguet insectarium platform; doi: 10.18167/infrastructure/00001) according to the regulations established by the Ethical and Animal Welfare Committee of the institution where the experiments were conducted (CIRAD, Montpellier, France), complying with the European legislation. The Baillarguet insectarium platform is a member of the National Infrastructure EMERG'IN and of the Vectopole Sud network (http://www.vectopole-sud.fr/) led by the joint units Intertryp (IRD, Cirad) and ASTRE (Cirad, INRAE). During the experiment, blood was taken from cows sheltered in the CIRAD animal facility according to a protocol approved under number APAFIS#1445‐2015081217184829v2 by the French Ministry of Research.

## Data Availability

The full rpoB nucleotide sequence for the R. lusitaniae R‐Om strain can be found in GenBank, with the accession number PP399358. The data set for the qPCR quantification of Rickettsia in both rifampin‐treated and untreated ticks, along with the command lines used for statistical and phylogenomic analysis, are accessible on GitHub: https://github.com/julien01A/Amoros-Fattar-2024-Rifampin

## APPENDIX 1

**Table A1:** Primers used in the qPCR assay for measuring *Rickettsia lusitaniae* R‐Om density.

Gene	Gene product	Target organism	Primers	Sequence (5′‐3′)
*OmAct2*	Actin 2	*Ornithodoros moubata*	Omou_actqF2	CGGTATTGCCGACCGTATGC
			Omou_actqR1	CTCCCTGTCCACCTTCCAGC
*gltA*	Citrate synthase	*Rickettsia lusitaniae* R‐Om	Rick‐gltA‐F‐Nw	GCTATTTGCAGGAGTGGGTTG
			Rick‐gltA‐R‐Nw	TCCGTTTAGGTTRATGGGCTT

**Table A2:** List of *Rickettsia* genomes used in this study.

*Rickettsia* group	Species	Strain	GenBank	Sequence available	Susceptibility to rifampin (cf. Table 1)
Spotted Fever Group	*R. aeschlimannii*	MC16	GCA_001051325	Complete genome	**R**
	*R. africae*	ESF‐5	GCA_000023005	Complete genome	**S**
	*R. amblyommatis*	An13	GCA_002078335	Complete genome	**U**
		GAT‐30V	GCA_000284055	Complete genome	**U**
	*R. argasii*	T170‐B	GCA_000965185	Complete genome	**U**
	*R. conorii*	A‐167	GCA_000261325	Complete genome	**S**
		Malish7	GCA_000007025	Complete genome	**S**
		Moroccan	AF076435	*rpoB* gene sequence	**S**
		ISTT CDC1	GCA_000263815	Complete genome	**S**
		ITTR	GCA_000257435	Complete genome	**U**
	*R. fournieri*	AUS118	GCA_900243065	Complete genome	**U**
	*R. gravesi*	BWI‐1	GCA_000485845	Complete genome	**U**
	*R. heilongjiangensis*	054	GCA_000221205	Complete genome	**U**
		CH8‐1DNA	GCA_009731545	Complete genome	**U**
	*R. honei*	RB	GCA_000263055	Complete genome	**S**
	*R. japonica*	YH	GCA_000283595	Complete genome	**S**
		Nakase	GCA_002357175	Complete genome	**U**
	*R. kotlanii*	HM‐2	GCA_030295365	Complete genome	**U**
		FLA‐4	GCA_030295345	Complete genome	**U**
	*R. massiliae*	AZT80	GCA_000283855	Complete genome	**R**
		Bar‐29	AF076436	*rpoB* gene sequence	**R**
		MTU1	AF076433	*rpoB* gene sequence	**R**
		MTU5	GCA_000016625	Complete genome	**U**
	*R. montanensis*	OSU 85‐930	GCA_000284175	Complete genome	**U**
	*R. parkeri*	Black Gap	GCA_018610945	Complete genome	**U**
		Portsmouth	GCA_000284195	Complete genome	**U**
	*R. raoultii*	IM16	GCA_001975185	Complete genome	**U**
		BIME	GCA_023674445	Complete genome	**U**
	*R. rhipicephali*	3‐7‐female6‐CWPP	GCA_000284075	Complete genome	**R**
		EH	GCA_026309315	Complete genome	**U**
	*R. rickettsii*	Brazil	GCA_000283955	Complete genome	**U**
		R	GCA_000831525	Complete genome	**S**
	*R. siberica subsp. sibirica*	246	GCA_000166935	Complete genome	**S**
		BJ‐90	GCA_000246715	Complete genome	**U**
	*R. siberica subsp. mongolitimonae*	HA‐91	GCA_000247625	Complete genome	**S**
	*R. slovaca*	13‐B	GCA_000237845	Complete genome	**S**
		D‐CWPP	GCA_000252365	Complete genome	**U**
	Rickettsia endosymbiont of *Proechinophthirus fluctus*	Pfluc4	GCA_020404545	Complete genome	**U**
Scapularis	*R. buchnerii*	ISO7	GCA_000696365	Complete genome	**U**
		PalLab	GCA_026723685	Complete genome	**U**
	*R. monacensis*	IrR/Munich	GCA_000499665	Complete genome	**U**
	*R. tamurae*	AT‐1	GCA_000751075	Complete genome	**U**
	*Rickettsia* endosymbiont of *Ixodes pacificus*	Humboldt	GCA_000965155	Complete genome	**U**
	*Rickettsia* endosymbiont of *Ixodes scapularis*	REIS	GCA_000160735	Complete genome	**U**
Transitional	*R. akari*	Hartford	GCA_000018205	Complete genome	**U**
	*R. asembonensis*	TH2014	GCA_019249195	Complete genome	**U**
		NMRCii	GCA_000828125	Complete genome	**U**
	*R. australis*	Cutlak	GCA_000284155	Complete genome	**U**
		Phillips	GCA_000273745	Complete genome	**S**
	*R. felis*	LSU‐Lb	GCA_000804505	Complete genome	**U**
		URRWXCal2	GCA_000012145	Complete genome	**S**
	*R. hoogstraalii*	CS	GCA_026309295	Complete genome	**U**
		RCCE3	GCA_000964845	Complete genome	**U**
	*R. lusitaniae*	R‐Om	This study	*rpoB* gene sequence	**S**
Typhus	*R. prowazekii*	BreinI	GCA_000367405	Complete genome	**S**
		Madrid E	GCA_000195735	Complete genome	**S**
	*R. typhi*	B9991CWPP	GCA_000277305	Complete genome	**U**
		Wilmington	GCA_000008045	Complete genome	**S**
Helvetica	*R. asiatica*	Maytaro1284	GCA_007989425	Complete genome	**U**
	*R. helvetica*	C9P9	GCA_000255355	Complete genome	**U**
Canadensis	*R. canadensis*	CA410	GCA_000283915	Complete genome	**U**
		McKiel	GCA_000014345	Complete genome	**U**
Adalia	*Rickettsia* endosymbiont of *Adalia bipunctata*	‐	‐	Complete genome	**U**
Bellii	*R. bellii*	An04	GCA_002078315	Complete genome	**U**
		OSU 85‐389	GCA_000018245	Complete genome	**U**
		RML 369‐C	GCA_000012385	Complete genome	**U**
		RML Mogi	GCA_000965045	Complete genome	**U**
	*Rickettsia* endosymbiont of *Bembidion lapponicum*	Blapp1	GCA_020404495	Complete genome	**U**
	*Rickettsia* endosymbiont of *Bembidion nr.*	Btrans1	GCA_020404375	Complete genome	**U**
	*Rickettsia* endosymbiont of *Columbicola hoogstraali*	Choog2	GCA_020404365	Complete genome	**U**
	*Rickettsia* endosymbiont of *Bemisia tabaci*	MEAM1	GCA_002285905	Complete genome	**U**
		wb	GCA_001707925	Complete genome	**U**
Meloidae	*Rickettsia* endosymbiont of *Pyrocoelia pectoralis*	Ppec13	GCA_020404425	Complete genome	**U**
Rhyzobius	*Rickettsia* endosymbiont of *Oxypoda opaca*	Oopac6	GCA_020881235	Complete genome	**U**
Torix	*Rickettsia* endosymbiont of *Gnoriste bilineata*	Gbili3	GCA_020881275	Complete genome	**U**
	*Rickettsia* endosymbiont of *Labidopullus appendiculatus*	Lappe4	GCA_020881075	Complete genome	**U**
	*Rickettsia* endosymbiont of *Pseudomimeciton antennatum*	Pante1	GCA_020881195	Complete genome	**U**
	*Rickettsia* endosymbiont of *Cimex lectularius*	RiClec	GCA_020410805	Complete genome	**U**
	Rickettsia endosymbiont of *Bryobia graminum*	Moomin	GCA_020881085	Complete genome	**U**
	*Rickettsia* endosymbiont of *Sericostoma sp.*	Ssp4	GCA_020404565	Complete genome	**U**
Candidatus Megeira (Outgroup)	*Candidatus Megaira* endosymbiont of *Mesostigma viride*	NEIS296	GCA_020410825	Complete genome	**U**
Orientia (Outgroup)	*Orientia tsutsugamushi*	Ikeda	GCA_000010205	Complete genome	**S**

Abbreviations: R, naturally resistant species and strains; S, susceptible; U, unknown.

**Table A3:** List of the putative genes only present in rifampin‐resistant *Rickettsia* species (without orthologs in susceptible species).

Putative gene^a^	Length (amino acids)	Gene localization	Position
Rrhi37F6_00877	192	Chromosome (CP003319)	785,232 to 785,811
		Chromosome (CP003342)	756,448 to 757,025
		Chromosome (CCER01000014)	149,448 to 150,023
Rrhi37F6_00879	62	Chromosome (CP003319)	784,432 to 784,599
		Chromosome (CP003342)	757,390 to 757,570
		Chromosome (CCER01000014)	150,656 to 150,841
Rrhi37F6_01547 *(CopG)*	76	Plasmid (CP003320)	1090 to 1317
		Plasmid (CP003343)	13,034 to 13,261
		Plasmid (CCER01000015)	13,131 to 13,358
Rrhi37F6_01549	59	Plasmid (CP003320)	51 to 227
		Plasmid (CP003343)	14,124 to 14,300
		Plasmid (CCER01000015)	14,233 to 14,409
Rrhi37F6_00531 *(ompA)*	97	Chromosome (CP003319)	1,086,010 to 1,086,225
		Chromosome (CP003342)	454,648 to 454,923
		Chromosome (CCER01000011)	199,573 to 199,863
Rrhi37F6_01548 *(ParA*)	213	Plasmid (CP003320)	437 to 1075
		Plasmid (CP003343)	13,276 to 13,914
		Plasmid (CCER01000015)	13,373 to 14,011
Rrhi37F6_00259	68	Chromosome (CP003319)	300,377 to 300,562 (1); 575,565 to 575,750 (2); 829,521 to 829,706 (3); 1,153,860 to 1,154,045 (4)
		Chromosome (CP003342)	210,128 to 210,316 (1); 386,792 to 386,980 (2); 712,238 to 712,426 (3); 980,467 to 980,655 (4); 1,259,577 to 1,259,765 (5)
		Chromosome (CCER01000003)	4707 to 4910 (1); 74,738 to 74,932 (2)
		Chromosome (CCER01000007)	196,482 to 196,676 (3)
Rrhi37F6_00257	65	Chromosome (CP003319)	301,032 to 301,208 (1); 576,220 to 576,411 (2); 895,439 to 895,630 (3); 1,153,199 to 1,153,390 (4)
		Chromosome (CP003342)	209,465 to 209,659 (1); 387,449 to 387,643 (2); 644,881 to 645,072 (3); 979,804 to 979,998 (4); 1,258,914 to 1,259,108 (5)
		Chromosome (CCER01000003)	74,080 to 74,274 (1)
		Chromosome (CCER01000007)	195,824 to 196,018 (2)
Rrhi37F6_01535	37	Plasmid (CP003320)	12,201 to 12,311
		Plasmid (CP003343)	1971 to 2081
		Plasmid (CCER01000015)	2104 to 2214
Rrhi37F6_01536	937	Plasmid (CP003320)	9041 to 12,007
		Plasmid (CP003343)	3384 to 5240
		Plasmid (CCER01000015)	2404 to 5214
Rrhi37F6_01542	55	Plasmid (CP003320)	4970 to 5147
		Plasmid (CP003343)	9530 to 9706
		Plasmid (CCER01000015)	10,787 to 10,951

*Note*. Accession numbers refer to genomes of R. massiliae strain AZT80 (CP003319), R. rhipicephali strain 3‐7‐female6‐CWPP (CP003342), and R. aeschlimannii (CCER01000003‐15).

^a^ provisional names.
